# Expert Opinion on Three Clinical Cases with a Common Urgent Problem: Urge Urinary Incontinence

**DOI:** 10.1155/2018/8567436

**Published:** 2018-10-16

**Authors:** Andrea Tubaro, John Heesakkers, Jean Nicolas Cornu, Dudley Robinson

**Affiliations:** ^1^Department of Urology, Sant'Andrea Hospital, “Sapienza” University, Via di Grottarossa 1035, Rome, 00189, Italy; ^2^Department of Urology, Radboud University Medical Center, Geert Grooteplein Zuid 10, Nijmegen 6525 GA, Netherlands; ^3^Department of Urology, Charles Nicolle University Hospital, University of Rouen, 1 rue de Germont, 76000 Rouen, France; ^4^Department of Urogynaecology, King's College Hospital, Denmark Hill, London SE5 9RS, UK

## Abstract

Urgency is the cornerstone symptom of overactive bladder (OAB) syndrome, which is associated with reduced health-related quality of life (HRQoL) and affects patients with different profiles. We report here three clinical pictures of OAB: a male patient with mixed lower urinary tract symptoms (LUTS), a young woman with comorbidities, and an elderly woman with mixed urinary incontinence. The aim is to analyze the specificities of these real cases, to discuss what would be the most appropriate management, and how treatment with fesoterodine, an antimuscarinic agent with key pharmacological properties, might meet the patients' expectations. Relevant and constructive messages are drawn: urgency, the cornerstone symptom, should be given special attention; fesoterodine is effective and well tolerated in the elderly; before switching to another medication consider increasing the dosage of fesoterodine; the major goal of initial therapy is to meet patient expectations; and involving the patient in the treatment plan increases her/his adherence.

## 1. Introduction 

Overactive bladder (OAB) syndrome covers several symptoms: urinary urgency, which is the cornerstone symptom [1], usually associated with urinary frequency and nocturia, and with or without urgency urinary incontinence (UUI). The term of OAB applies if there is no proven infection or other obvious pathology [2, 3]. OAB is correlated with reduced health-related quality of life (HRQoL) and affects patients with different profiles. Patients with OAB may be children, premenopausal and postmenopausal women, men with benign prostate hypertrophy (BPH), or frail elderly patients. Their symptoms are known to be bothersome and treatable [4]. Patients have different needs and also specific expectations, which are sometimes challenging for the physician in charge, but must be set right. Gynecologists often care for women with urinary symptoms of urgency and frequency, but they may not always be recognized as OAB.

We report three main clinical pictures of OAB: a male patient presenting with lower urinary tract symptoms (LUTS), including urgency; a young woman with comorbidities; and an elderly woman with mixed urinary incontinence, with the aim of highlighting the specific features of these real-life cases, discussing the most appropriate management, and assessing how fesoterodine treatment may meet these patients' expectations.

Fesoterodine possesses four key pharmacological properties [5–8]. Firstly, it is immediately and completely metabolized into its active form, the 5-hydroxymethyl tolterodine (5-HMT) by ubiquitous plasma esterases. Thus, its activation is not affected by genetic variability in hepatic enzymes [5]. This may explain the dose-response relationship observed clinically. Secondly, fesoterodine has a very low risk of crossing the blood brain barrier, and has no significant effect on cognitive function, which has been confirmed in clinical trials [6]. Thirdly, it possesses a well-balanced affinity ratio on detrusor M2 and M3 receptors, which explains its further actions on contraction and on relaxation, increasing the bladder capacity [9]. Finally, the 7–9-hour half-life of fesoterodine allows a single daily dose to be taken, avoiding the risk of accumulation of the active ingredient [10].

As mentioned in the product specifications (Toviaz® SPCs) [11], two doses, 4 and 8 mg, are available. The recommended starting dose is 4 mg once daily. Fesoterodine 8 mg has proven its superiority compared to 4 mg in reducing UUI episodes in a head-to-head comparison in OAB patients; the 8 mg dose was also more effective in controlling frequency and nocturia [12].

## 2. First Case: Mr. LUTS and OAB

The first case reported the history of “Mr. LUTS and OAB”, who is 72 years of age, has had LUTS for five years, has a weak and variable stream, complains of painful micturition if he waits too long between micturitions, and also of frequency, urgency, and nocturia. There were no signs of bladder outlet obstruction or other identified etiology. He has been diagnosed with hypertension and type 2 diabetes. He takes several medications: a beta-blocker, a selective AT1-subtype angiotensin II receptor antagonist, a calcium antagonist, and an oral biguanide antihyperglycemic agent. His International Prostate Symptom Score (IPSS) is 15, and the QoL question was scored as 4. Digital rectal examination revealed a slight increase in the size of the prostate, with an estimated volume of 50–60 ml. He has been offered transurethral resection of the prostate (TURP) but declined. The European Association of Urology (EAU) guidelines [16] state that prostate-specific antigen (PSA) should be tested if a diagnosis of prostate cancer will change management or if it assists in the treatment and/or decision-making process; his PSA was within the normal range, at 2.6 ng/ml. His IPSS value for storage symptoms was higher than his score for voiding symptoms. He was prescribed an alpha blocker (tamsulosin) as a first-line treatment. Six weeks later, at the next visit, he reported a slight improvement in frequency and stream, with no change in nocturia or urgency, and some ongoing rare UUI episodes and dysuria. His QoL score, which was linked to urgency, was not improved. Uroflowmetry was performed and showed a Qmax of 11 ml/s, a voiding volume (VV) of 140 ml, and a postvoiding residual (PVR) of 45 ml. Transrectal ultrasound confirmed an enlarged prostate (68 ml).

“Mr. LUTS and OAB” was offered a combination treatment with tamsulosin and dutasteride to try to control his symptoms and reduce the weight of his prostate. Three months later, he came back for a follow-up visit and declared he had stopped dutasteride after two weeks because of erectile dysfunction. He was still complaining of nocturia and urgency. The control of PVR indicated a residue lower than 100 ml.

A frequency-volume chart (FVC) that collects the time and volume of micturitions can be a useful tool to obtain a real assessment of nocturia. Van Haarst et al. [17] analyzed two different ways of measuring nocturia and showed that in 50% of the patients the IPSS nocturia score was found to be higher than nocturia calculated from the FVC, indicating that the IPSS overestimates nocturia in most patients.

The analysis of the patient's voiding diary revealed a night-time urine output of 800–950 ml and a 24-hour urine output of 2,600–2,900 ml. Therefore, an adjustment of fluid intake was advised. The clinical picture led to the prescription of a combination treatment of tamsulosin 0.4 mg plus fesoterodine 4 mg, and the patient agreed to enroll in a telephone and ambulatory clinic follow-up program.

One point of concern was nocturia. Weiss et al. [18] showed that significant improvements were observed with fesoterodine compared to placebo in the number of micturition-related nocturnal urgency episodes (–1.28 versus –1.07,* p* = 0.003), in the number of nocturnal micturitions per 24 hours (–1.02 versus –0.85,* p* = 0.011), and in the nocturnal frequency urgency sum, defined as the sum of Urinary Sensation Scale (USS) ratings recorded for all nocturnal micturitions (–4.01 versus –3.42,* p* < 0.003). Additionally, HRQoL measures were improved with fesoterodine.

A further six weeks later, the patient came back with no change in nocturia, a slight improvement in urgency, and complained of minimal dry mouth. Consequently, the fesoterodine dose was increased to 8 mg per day and the patient was kept in the follow-up study. His IPSS improved to 10 and his QoL score on the IPSS improved to 2.

### 2.1. Discussion

This interesting case highlights important clinical aspects leading to useful conclusions:

- The patient related his symptoms to BPH and was at the beginning treated as a BPH patient only. However, as storage symptoms and nocturia were also present, the patient needed a customized (nonfixed) combination.
- In clinical studies, fesoterodine has demonstrated efficacy on nocturia unrelated to nocturnal polyuria; nocturnal polyuria should be managed by reducing the urine output at night.
- Urgency was actually the major cause of the QoL deterioration but was treated last.
- An appropriate follow-up program kept the patient on treatment.

## 3. Second Case: Mrs. Young OAB

The second case refers to a 48-year-old woman, a busy manager with a history of depression and sleep disturbance. She has had three terminations of pregnancy and one delivery by cesarean section. She smokes approximately ten cigarettes per day and has high cholesterol serum levels. She takes several medications: a selective serotonin reuptake inhibitor (escitalopram), two benzodiazepines (delorazepam and clonazepam), and a statin. She reports a four-year history of urinary symptoms: daily UUI episodes, mild stress urinary incontinence (SUI), and two episodes of nocturia per night. She wears pads every day. The urology consultation revealed some degree of pelvic pain, especially during vaginal examination. The urine dipstick was negative and there was no PVR. No specific causes of the symptoms such as urine tract infection were identified. The patient also complained of mild dyspareunia and occasional constipation. The urine culture turned out to be sterile, with no blood in urine, and the pelvic ultrasound scan and urine cytology were also negative. The cystoscopy, which was performed as a result of the presence of storage symptoms and to rule out a bladder tumor in this current smoker, was normal.

In OAB patients, it is of utmost importance to consider all comorbidities. Anxiety and depression may play a role, feeding a vicious circle. Moreover, medications to treat neurological or psychiatric disorders can influence OAB and be responsible for side effects [19, 20]. Gastrointestinal disorders are frequently associated with OAB, such as constipation in this case, but patients rarely raise the topic. An overlap exists between irritable bowel syndrome and OAB [21].

The patient was prescribed a *β*3 agonist, pelvic floor muscle training (PFMT) and bladder retraining. Four months later, she noticed some degree of improvement, but had stopped the treatment as she felt that she had no time for PFMT. She was not compliant with the bladder drill either, and soon stopped the *β*3 agonist because she did not sense any real improvement. She also felt that she did not have the time to complete a bladder diary. She was prescribed fesoterodine 8 mg for three months. In parallel, her general practitioner asked for vaginal and urethral culture swabs, which were negative. After three months, her urinary urgency improved, but she said that the few remaining episodes of urgency were “killing her life” and that she did not want to be on pills for her whole life. Therefore, she refused to continue the treatment and requested an “easy fix”. Her reaction highlights the need for careful consideration of the consequences of incontinence in terms of QoL. A publication from Vaughan et al. [22] reported that OAB and incontinence synergize to reduce QoL, especially in the domains of sleep, elimination, usual activities, discomfort, distress, vitality, and sexual activity.

Consistent efficacy on urgency symptoms with a significant decrease in UUI and urgency episodes has been reported with fesoterodine at doses of 4 and 8 mg compared to placebo (Table 1) [12, 13, 23, 24]; however, some patients may react differently. Patient satisfaction is an important driver of treatment success [14]. Patient expectations should be considered carefully in the context of OAB management. The achievement of patients' goals was measured in the Study Assessing FlexIble-dose fesoterodiNe in Adults (SAFINA study) [15], a 12-week multicenter open label study with 331 OAB adults, using the Self-Assessment Goal Achievement (SAGA) questionnaire. Fesoterodine treatment resulted in 81.3% of patients declaring that their goals were “somewhat achieved/achieved” or that the result “exceeded/greatly exceeded their expectation”.

Our case patient had very specific expectations; she refused to have an implant (neuromodulation), saying “I'm not going to be an android!” She accepted botox injections, and so a first set of injections was performed under local anesthesia. She found the injections “a little painful” and “a big annoyance”, but at the one-month follow-up visit after botox injection she reported no more UUI episodes and an improvement in frequency and the number of urgency episodes, as well as in QoL. Even though she stated that she did not like the idea of being a patient for the rest of her life, she accepted subsequent injections.

### 3.1. Discussion

The clinical points that can be learned from this case are as follows:

- All OAB cases are different, and a thorough evaluation is mandatory to adequately address each case.
- It is important to assess other aspects, such as functional and psychological disorders that may influence symptoms, and to consider nonneurogenic OAB as a multifactorial disease.
- The major goal of initial therapy is to meet the patient's expectations regarding the reason for their visit, to improve their satisfaction, and their QoL.
- Due to fesoterodine's characteristics and flexible dosage, improvement of symptoms and achievement of the patients' goal are usually high with this medication.
- When patients have specific requirements, all options should be discussed and the patient's agreement obtained.
- A customized approach is a crucial factor for treatment success.
- OAB management should be personalized; beware of a simplistic application of a standardized treatment algorithm.

## 4. Third Case: Mrs. Mixed Urinary Incontinence

The third case concerns a 73-year-old retired schoolteacher who had had two children, born by forceps-assisted vaginal delivery. She complained of ten episodes of daytime frequency with small frequent voids, a constant desire to void, and three nocturia episodes per night, resulting in poor sleep. More recently, things had deteriorated with a sudden urge to void and occasional urinary incontinence with exercise. She changed pads three times a day and complained of superficial dyspareunia. She took medication (doxazosin and furosemide) for well-controlled hypertension but did not take hormone replacement therapy. She complained of mild constipation and had had three lower urinary tract infections (UTIs) in the last 12 months. During her consultation with her general practitioner, she emphasized that urgency was the most important problem for her. She was referred to a gynecologist, who confirmed a subjective diagnosis of mixed urinary incontinence with superficial dyspareunia and vulval discomfort. The physical examination detected a marked urogenital atrophy, a grade I uterine prolapse, a grade I cystocele, and grade I rectocele. She was initially prescribed vaginal estrogen, as supported by evidence from Cochrane reviews [25, 26], and referral to a urogynecologist was considered.

The urogynecologist confirmed the mixed urinary symptoms with urgency and UUI, SUI, recurrent UTIs, and mild constipation. The clinical examination also confirmed urogenital atrophy, demonstrable SUI, and a urogenital prolapse without evidence of neuropathy or specific etiology of the symptoms.

The PVR was found to be 95 ml, urinalysis was negative, and flexible cystoscopy was normal. The patient noticed an improvement of vulval discomfort and dyspareunia with local estrogen therapy. The physician provided lifestyle advice, prescribed PFMT training and oxybutynin 5 mg three times a day, and undertook a medication review. The alpha blocker doxazosin, which may aggravate the symptoms of SUI, was switched to an angiotensin II receptor antagonist, losartan, and furosemide was discontinued. The International Consultation on Incontinence Modular Questionnaire (ICIQ) and a bladder diary were completed. They showed that the patient leaked a moderate amount of urine several times a day, generating moderate bother. Incontinence occurred before she could get to the toilet and was also associated with exercise, coughing, and sneezing. She complained of ten frequency episodes per day, three nocturia episodes with moderate bother, and had a total VV at 2,190 ml. The night volume was 490 ml, with no evidence of nocturnal polyuria. This led to the symptomatic diagnosis of mixed incontinence, which is an important common symptom and is particularly difficult to treat. It has been defined by Haylen et al. [27] as being “the complaint of involuntary loss of urine associated with urgency and also with effort or physical exertion or on sneezing or coughing”.

After six weeks, the patient returned and reported that she had taken oxybutynin for six weeks and then stopped due to persistent dry mouth and worsening constipation. She had also performed bladder retraining and PFMT for two weeks and then stopped. She noticed a mild improvement in urgency and UUI, but no change in nocturia, and her SUI was still troublesome. The urogynecologist suggested switching to fesoterodine 4 mg once a day and explained that she could increase the dose if required [13, 28, 29].

What are the reasons to consider using fesoterodine, here as a second line therapy, in a 73-year-old elderly woman? Fesoterodine is the only OAB medication classified as B (Beneficial) by the Fit fOR The Aged (FORTA) classification [30]. LUTS-FORTA results from a systematic review of drugs treating LUTS in the elderly in order to evaluate their efficacy, safety, and tolerability. Darifenacin, mirabegron, oxybutynin ER, solifenacin, tolterodine, and trospium were classified as FORTA C (Questionable; Caution), while oxybutynin IR and propiverine were classified as FORTA D (Avoid; Do not). This classification suggests that fesoterodine may have better efficacy and tolerability in elderly patients.

Moreover, the combination of fesoterodine with vaginal estrogen may act synergistically. Chugtai et al. [31] have studied the results of combining fesoterodine with vaginal estrogen and showed that this combination improved OAB symptoms and sexual function in postmenopausal women, and importantly, produced greater improvement in QoL.

At 12 weeks the urogynecologist reviewed her clinical picture. Fesoterodine 4 mg was well tolerated and there was no need for a dose increase, her urgency and urgency incontinence symptoms were improved, and the number of voids per day had decreased to seven, but remained at two per night. Her symptoms of urogenital atrophy and dyspareunia had improved and there was no reported UTI. Constipation improved with dietary advice and possibly by stopping oxybutynin as well. While her urogenital prolapse remained asymptomatic, her SUI remained troublesome. With regard to adverse effects, the fact that patients cope better with dry mouth with fesoterodine compared to oxybutynin may lie in the selectivity of 5-HMT for the bladder over the salivary glands. An* in vitro* and* in vivo* study, using radioligand binding, has demonstrated that oxybutynin has a threefold higher affinity for the salivary glands over the bladder [32] and therefore has a greater propensity to induce dry mouth.

At the 24-week visit, the urogynecologist's review was in favor of continuing with fesoterodine 4 mg since the patient's urgency and UUI were much improved. However, there was still some SUI limiting her physical activity. The urodynamic investigations performed while the patient was on fesoterodine showed a cystometric capacity of 500 ml, no evidence of detrusor overactivity, moderate urodynamic SUI, and no evidence of voiding dysfunction. Hence, as suggested by the 6th International Consultation on Incontinence [33], the urogynecologist discussed the possibility of surgery for SUI and continued the current pharmacological treatment.

### 4.1. Discussion

This clinical case illustrates some important take-home messages:

- It is important to treat the most bothersome symptoms first.
- In case of mixed urinary symptoms, there is a need to look for a possible urogenital prolapse; this particular patient had demonstrable genital prolapse but did not find it troublesome.
- Fesoterodine is effective and well tolerated in the elderly, and is rated B (beneficial) by the LUTS-FORTA classification.
- Vaginal estrogen is effective in the management of urogenital atrophy and recurrent UTIs.
- There is evidence to support the combined use of estrogen and antimuscarinics.
- Nocturia secondary to nocturnal polyuria does not respond to antimuscarinic medication and should benefit from lifestyle advice and consideration of desmopressin treatment.

Finally, the conclusions were that, firstly, the cornerstone symptom of OAB, urgency, is very common, and that many OAB patients may not be diagnosed appropriately. Secondly, when urgency is diagnosed, the use of antimuscarinics should be considered. Thirdly, fesoterodine has the benefit of flexible dosing and can be titrated accordingly; therefore, before switching to another medication, increasing the dose for further efficacy should be considered. Finally, the patient should not be considered as a passive subject but as a real actor in the treatment process; involving her/him in the treatment plan increases her/his adherence and the improvement of treatment outcomes.

## Figures and Tables

**Table 1:** Percentage decrease in urinary urgency incontinence and urgency episodes according to different studies with fesoterodine at 4 and 8 mg.

	**UUI decrease ** **(**%**) 4/8 mg**	**Urgency episode decrease (**%**) 4/8 mg**
Chapple, 2007 [13]	80.8/87.5	17.6/19.1
Chapple, 2014 [12]	74.4/79.5	37.8/45.5
Kaplan, 2010 [14]	75.0	43.3
Dubeau, 2014 [15]	69.3	41.5
